# Mass production of poly(ethylene glycol) monooleate-modified core-shell structured upconversion nanoparticles for bio-imaging and photodynamic therapy

**DOI:** 10.1038/s41598-019-41482-w

**Published:** 2019-03-26

**Authors:** Xingyuan Zhang, Zhao Guo, Xiao Zhang, Linji Gong, Xinghua Dong, Yanyan Fu, Qing Wang, Zhanjun Gu

**Affiliations:** 1 Institute of Nano Engineering, College of Civil Engineering and Architecture, Shandong University of Science and Technology, Qingdao, 266590 China; 2 Key Laboratory for Biomedical Effects of Nanomaterials and Nanosafety, Institute of High Energy Physics, Chinese Academy of Sciences, Beijing, 100049 China; 3 University of Chinese Academy of Sciences, Beijing, 100049 China; 4 School of Material Science and Engineering, Shandong University of Science and Technology, Qingdao, 266590 China; 5 State Key Lab of Transducer Technology, Shanghai Institute of Microsystem and Information Technology, Chinese Academy of Sciences, Shanghai, 200050 China

## Abstract

Developing robust and high-efficient synthesis approaches has significant importance for the expanded applications of upconversion nanoparticles (UCNPs). Here, we report a high-throughput synthesis strategy to fabricate water-dispersible core-shell structured UCNPs. Firstly, we successfully obtain more than 10 grams core UCNPs with high quality from one-pot reaction using liquid rare-earth precursors. Afterwards, different core-shell structured UCNPs are fabricated by successive layer-by-layer strategy to get enhanced fluorescence property. Finally, the hydrophobic UCNPs are modified with poly(ethylene glycol) monooleate (PEG-OA) though a novel physical grinding method. On the basis of mass-production, we use the as-prepared PEG-UCNPs to construct an 808-nm stimuli photodynamic therapy agent, and apply them in cancer therapy and bio-imaging.

## Introduction

In recent years, rare-earth upconversion nanoparticles (UCNPs) have attracted much attention due to their unique optical properties, such as large anti-stokes shifts, high signal-to-noise ratio, and remarkable photo- and chemical stability^1–7^. Among all kinds of host matrixes of UCNPs, sodium lanthanide tetrafluoride (NaLnF_4_) nanocrystals, especially the hexagonal structure crystals (β-phase NaLnF_4_), are the most studied host materials due to the low phonon energies and the high efficiency of energy transfer^8–16^. Uniform nanocrystals of β-phase NaLnF_4_ have been produced through thermal decomposition method using various rare-earth precursors^17–21^. Unfortunately, only few researchers have developed mass-production strategy^22,23^. Developing a novel synthesis method to massively product high quality NaLnF_4_ nanocrystals is highly imperative.

Since thermal decomposition precursors play an important role in synthesis, selecting an appropriate lanthanide precursor would help expand production. The most commonly used precursors, such as lanthanide chlorides (LnCl_3_) and trifluoroacetates (Ln-TFA), have some difficulties in practice, e.g., the unsatisfied solubility. Methanol is usually required as solvent. However, the extra procedure of removing methanol makes it environmental-unfriendly, and even no feasibility in mass-production. Moreover, due to the presence of large amounts of water in LnCl_3_, it may lead to integration of water into the crystal structure of the UCNPs which can decrease the upconversion quantum yield^24^. Alternatively, using lanthanide trifluoroacetates as precursor would yield a large amount of toxic fluorinated by-products. Therefore, new nontoxic and soluble precursors should be investigated.

Currently, it has been reported that metal-surfactant complex, such as rare-earth oleates (Ln-OA), would make an effective precursor for synthesizing nanoparticles^25–28^. Our research group has successfully realized the controllable synthesis of β-phase NaLnF_4_ crystals using Ln-OA precursors, and pointed out the optimized synthetic parameters^29^. Ln-OA could be easily dissolved in reaction media, which would make the procedure facile compared with the other rare-earth precursors. This unique advantage provides a chance to expand the production of UCNPs and further accelerate the process of practicality.

Using liquid precursors could also contribute to the synthesis of core-shell structured UCNPs. Core-shell structure is of great importance to the optical properties of UCNPs^24,30–35^. Additional shell layers of different materials or compositions endow UCNPs either improved the features or new interesting properties (e.g., the pure host material inert shell could enhance luminescence intensity and the neodymium shell could regulate the excitation wavelength)^36–40^. In some cases, the accurate tuning requires more than two coated shells^41–44^. The existing strategy could only synthesize one layer during one-pot reaction. To make up the deficiencies, successive layer-by-layer (SLBL) strategy was introduced to synthesize multi-shell structured UCNPs instead of tedious multi-cycle batch operation^45,46^. SLBL strategy usually required successive injection of raw materials, which coincidently matched the easily soluble character of Ln-OA precursors. Thus, we hoped that using Ln-OA precursors would make SLBL strategy more flexible and manoeuvrable.

In this work, we developed a novel high-throughput method to synthesize poly(ethylene glycol) monooleate (PEG-OA) modified multi-shell structured upconversion nanoparticles (PEG-UCNPs), and then utilized them for photodynamic therapy (PDT). By optimizing the reaction parameters, we obtained more than 10 grams of products with uniform size and morphology in a single reaction. Afterwards, we realized the successful mass-production of multi-shell structured UCNPs using SLBL strategy and liquid Ln-OA precursors. Three different core-shell structural UCNPs with the enhanced upconversion luminescence (UCL), optional excitation source or orthogonal excitations-emissions properties were fabricated. Furthermore, gram quantities of hydrophilic UCNPs with PEG-OA modification were gained through a novel grinding method. Finally, a blue light excited photosensitizer, Hypocrellin A (HA), was loaded on the surface of PEG-UCNPs to construct a PDT platform for simultaneous bio-imaging and PDT in cancer.

## Results and discussion

### High-throughput synthesis of β-phase core NaYF_4_ nanocrystals

**Figure 1:**
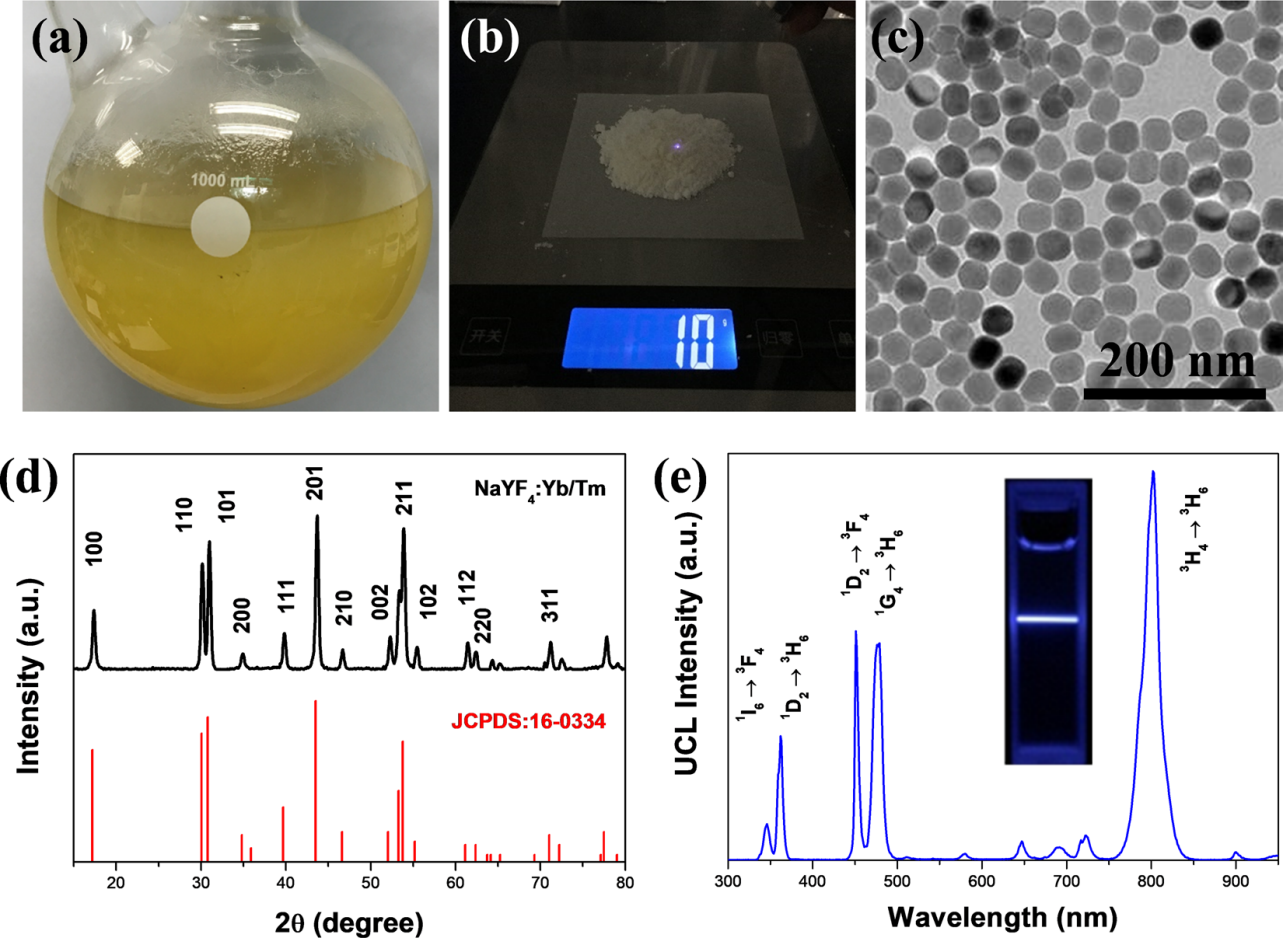
(**a**) The digital photo of the reaction apparatus. (**b**) The total weight of the obtained product was about 10 grams. (**c**) TEM images of NaYF_4_:Yb/Tm nanoparticles that were synthesized using Ln-OA. (**d**) XRD patterns of the prepared UCNPs and the standard PDF card. (**e**) Fluorescence spectrum of the as-prepared UCNPs under the excitation of 980-nm laser. Inset: UCL photo of UCNPs in cyclohexane. (2 mg mL^−1^, laser power: 1 W cm^−2^).

To achieve mass-production of uniform-sized monodisperse UCNPs, the liquid Ln-OA precursors were firstly prepared via the reaction of lanthanide nitrate and sodium oleate. After that, large quantities of UCNPs were obtained through amplifying the reaction volume (Fig. 1a). Due to the increased amount of reagents, the reaction time during thermal decomposition process should be modified. Taken the synthesis of NaYF_4_:Yb/Tm as an example, the scanning electron microscopy (SEM) images showed that heterogeneous UCNPs were obtained until the reaction time increased to 2 hours (Fig. S1). The X-ray diffraction (XRD) patterns confirmed that the crystallinity of the as-prepared UCNPs became better with the increase of reaction time (Fig. S2). After optimizing the reaction time, mass-production of NaYF_4_:Yb/Tm nanoparticles were performed. As shown in Fig. 1b, the total weight of the obtained products was about 10 grams and the yield was calculated to be about 67.8%. Transmission electron microscope (TEM) image in Fig. 1c showed that the uniform-sized and monodisperse nanoparticles were fabricated. Although the previous studies have reported some mature methods to synthesize UCNPs using LnCl_3_ as precursors, none of them were favourable in mass-production reaching to 10 grams. The poor solubility of LnCl_3_ in reaction solvent (OA and ODE) usually requires methanol to assist dissolve. However, in mass-production, removing such large number of toxic organics is not feasible and safe. More importantly, we verified that the residual methanol had dramatic effect on the uniformity of the produced UCNPs (Fig. S3). Similarly, another widely used precursor Ln-TFA was also unsuitable in mass-production due to its toxic by-products. From these results, we pointed that the metal-surfactant complex could be the most suitable precursor for the synthesis of gram-scaled high-quality UCNPs. As shown in Fig. 1d, the as-prepared UCNPs exhibited pure β-phase NaYF_4_ crystal form, which indicated a good crystallinity. As one of the most efficient host matrices for UCL, the emission spectrum of the as-prepared UCNPs was measured under 980-nm laser excitation. Four strong emission peaks were observed at 345 nm, 361 nm, 450 nm and 475 nm, which represented ^1^I_6_ → ^3^F_4_, ^1^D_2_ → ^3^H_6_, ^1^D_2_ → ^3^F_4_, ^1^G_4_ → ^3^H_6_ and ^3^H_4_ → ^3^H_6_ transition of Tm ions, respectively (Fig. 1e). Small size Tm ions doped UCNPs can also be obtained at low reaction temperature (Fig. S4). Besides, Er ions doped green-emitted UCNPs could also be obtained with uniform size, good crystallinity and strong UCL emission using the same method (Fig. S5). The upconversion quantum yield of Er-doped UCNPs from mass-production was ~0.14% at a power density of 10.0 W/cm^2^, which was a litter higher than the previous report^22,24^. The quantum yield of traditional small-scale synthesis is 0.15% with the same power density and there is no significant difference compared to UCNPs from mass-production. The mass-production strategy described here avoids using water or alcohols as solvents, and thus prevent the obtained UCNPs from quenching effects or low quantum yield. We believe that this strategy has the potential to be applied in pilot scale test, and finally utilized in industrial applications.

### Synthesis of different core-shell structured UCNPs via successive layer-by-layer method

**Figure 2:**
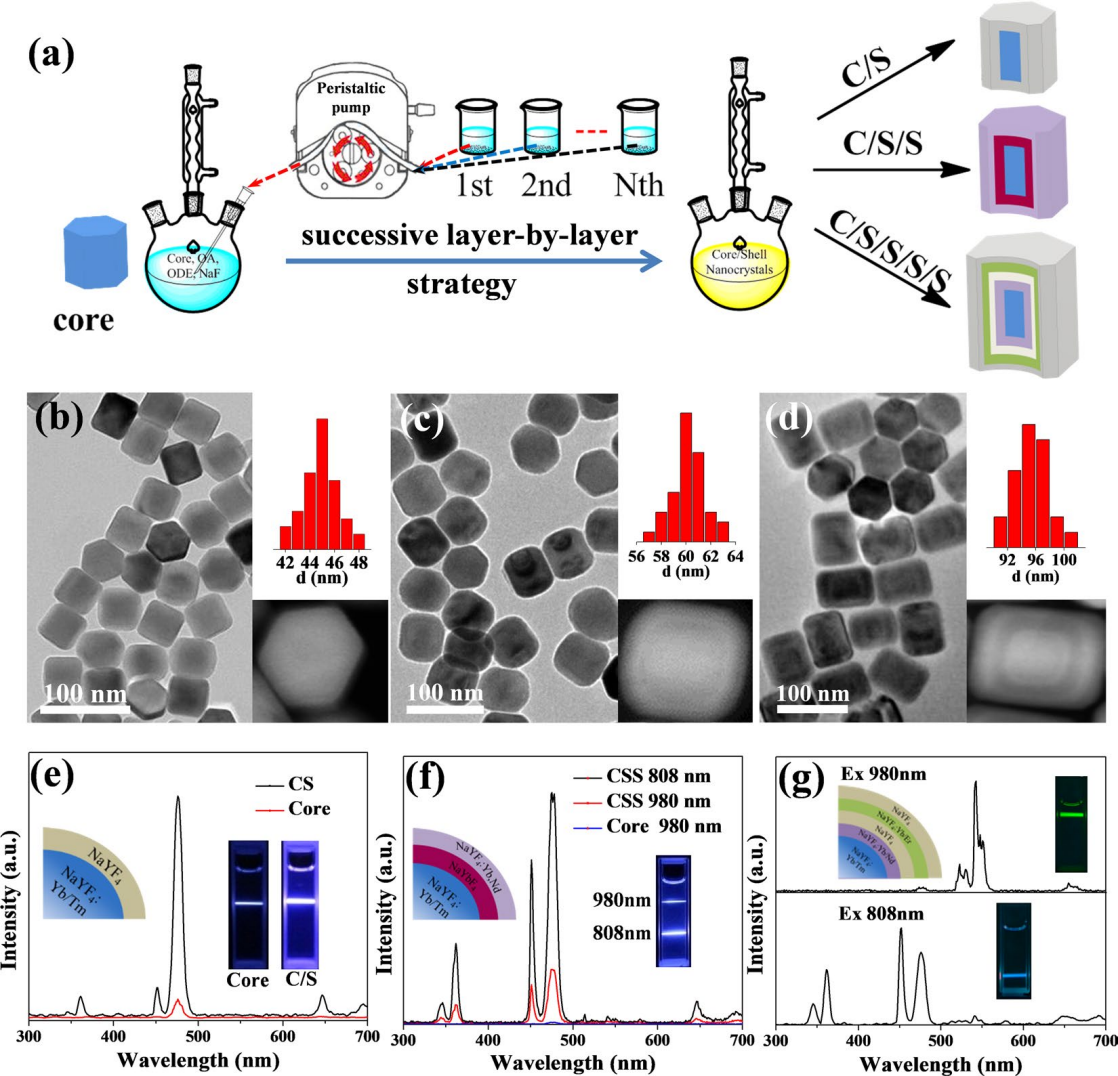
(**a**) Successive layer-by-layer (SLBL) synthetic procedure for three different structured UCNPs. (**b**–**d**) TEM images, particle size distribution and High Angle Annular Dark Field (HAADF) images of CS-UCNPs, CSS-UCNPs and MS-UCNPs. (**e**–**g**) Fluorescence spectra of the UCNPs under the excitation of 980-nm and 808-nm (except CS-UCNPs). Inset: UCL photos and schematic diagrams of different structured UCNPs.

To obtain advanced UCL properties, core-shell structured UCNPs were synthesized using SLBL method. As shown in Fig. 2a, a certain amount of core NaYF_4_:Yb/Tm nanoparticles and sodium fluoride (NaF) were dispersed in oleic acid (OA) and 1-octadecene (ODE), and then heated to the reaction temperature. The shell precursors of Ln-OA were slowly injected during the reaction using a peristaltic pump. By altering the lanthanide precursors, we successfully synthesized three different kinds of core-shell structured UCNPs with enhanced optical properties: i) core-shell NaYF_4_:Yb/Tm@NaYF_4_ (CS-UCNPs) have strong UCL; ii) core-shell-shell NaYF_4_:Yb/Tm@NaYbF_4_@NaYF_4_:Yb/Nd (CSS-UCNPs) could be excited by both 980-nm and 808-nm laser; iii) multi-shell NaYF_4_:Yb/Tm@NaYF_4_:Yb/Nd@NaYF_4_@NaYF_4_:Yb/Er@NaYF_4_ (MS-UCNPs) could independently emit two different colours fluorescence under two different excitation.

The reaction time and temperature played important roles in shell coating procedure. To simplify the problem, we kept the reaction time consistent with the total precursor injection time (1 mmol/h, the reaction time is 1 h for each shell). Thus, we investigated the influence of reaction temperature in shell coating process. The SEM images in Fig. S6 showed that higher reaction temperature were beneficial to obtain pure β-phase crystals. As shown in Fig. S7, the XRD pattern demonstrated the same conclusion where the appropriate reaction temperature was about 320 °C. Using this parameter, we further synthesized CS-UCNPs to achieve brighter fluorescence. As shown in Fig. 2b, the obtained NaYF_4_:Yb/Tm@NaYF_4_ nanoparticles exhibited hexagonal morphology with an average diameter of 45 nm and the thickness of shell is about 7 nm. It is well known that the inert NaYF_4_ shell could protect the doped luminescent ions from quenching arising from surface ligands or solvents. The UCL intensity of core-shell structured NaYF_4_:Yb/Tm@NaYF_4_ nanoparticles increased at least 10 fold than the core-only NaYF_4_:Yb/Tm nanoparticles (Fig. 2e).

The appropriate design of coating shells could also help regulate the excited wavelength of UCNPs. Herein, we synthesized CSS-UCNPs, which could be excited by 808-nm laser instead of the most frequently used 980-nm laser, due to the absorbance of Nd^3+^ ion (Fig. S8). The TEM images showed that the average size of the CSS-UCNPs was about 60 nm (Fig. 2c). Moreover, the core-shell-shell structure was clearly discerned in High Angle Annular Dark-field (HAADF) TEM image where the brighter regions represented Yb element and the darker parts corresponded with Y element. The thickness of shells are 7 nm of NaYF_4_ and 6 nm of NaYF_4_:Yb/Nd. The UCL properties of the as-prepared CSS-UCNPs were examined with the excitation of 808-nm and 980-nm lasers with the same power density. As shown in Fig. 2f, in both cases, the CSS-UCNPs emitted strong blue light. Interestingly, the UCL intensity of CSS-UCNPs under 808-nm excitation was much higher than that of under 980-nm excitation, which might be attributed to the relatively higher extinction coefficient of Nd^3+^ ions rather than Yb^3+^ ions. Besides, the use of 808-nm laser as excitation source instead of 980-nm laser could efficiently increase the penetration depth in biological tissues and reduce the unwanted overheating effect. This character made 808-nm excited CSS-UCNPs with great potential in biomedical applications.

The most attractive feature of SLBL strategy is to obtain multi-shell structured UCNPs by simply adjusting the ratio of different Ln-OA precursors. In this work, we successfully synthesized NaYF_4_:Yb/Tm@NaYF_4_:Yb/Nd@NaYF_4_@NaYF_4_:Yb/Er@NaYF_4_ multi-shell structured nanoparticles (MS-UCNPs) through SLBL method. The TEM image in Fig. 2d showed the uniform hexagonal prism nanoparticles with an axis of about 75 nm and a length of about 96 nm while the multi-shell structure could be observed in HAADF image. Every shell layer has good homogeneity, and the thickness of each shell is about 9 nm, 7 nm, 8 nm and 8.5 nm (Fig. S9). The UCL emission properties of MS-UCNPs were further investigated. The as-prepared MS-UCNPs could emit blue light under 808-nm irradiation as well as green light under 980-nm irradiation (Fig. 2g). In this multi-shell structure, the Tm^3+^ and Nd^3+^ ions were connected by Yb^3+^ ions whereas the Er^3+^ and Nd^3+^ ions were separated by a NaYF_4_ interlayer (Fig. S10). On excitation at 808-nm, the photon energy was absorbed by Nd^3+^ ions, then transferred to Yb^3+^ and finally diverted to Tm^3+^ ions to produce UV and blue emission. Upon 980-nm irradiation, Yb^3+^ ions in outer layers absorbed most photon energy and directly activated Er^3+^ ions nearby to generate green emission. This independent orthogonal excitation-emission feature made MS-UCNPs suitable for multi-dimensional security designs and imaging-guided multi-modal therapy.

Taken these results together, we have fabricated various kinds of core-shell UCNPs to obtain enhanced optical properties. The liquid feature of Ln-OA played a significant role in SLBL method. Combination of Ln-OA and SLBL strategy offered several important advantageous features including the feasibility and convenience in synthesis of complex structured UCNPs.

### Surface modification

To apply in biomedical applications, the hydrophobic UCNPs should be modified with hydrophilic ligands so that they could be dispersed in aqueous solution. Utilization of amphiphilic molecules, such as polyethylene glycol derivatives, is a well-established strategy for surface modification of UCNPs^30,47,48^. The hydrophobic chain of PEG-OA has hydrophobic interactions with oleic acid molecule bonding on the surface of UCNPs while the hydrophilic chain exposed outside. This makes the PEG-coated UCNPs water-dispersible. The traditional method is to disperse UCNPs and ligands in a mix of water and organic solvents, and then sonication. The limitation is that it can hardly increase the reaction scale because UCNPs with high concentration are easy to aggregate. Herein, we established a novel large-scaled method to modify UCNPs by physical grinding. Our method is to use physical effects to press PEG-OA and UCNPs together. During the process, the speed of ball grinding mill is the key factor. The UCNPs can be fully mixed with PEG-OA and not be broken down in optimal speed.

**Figure 3:**
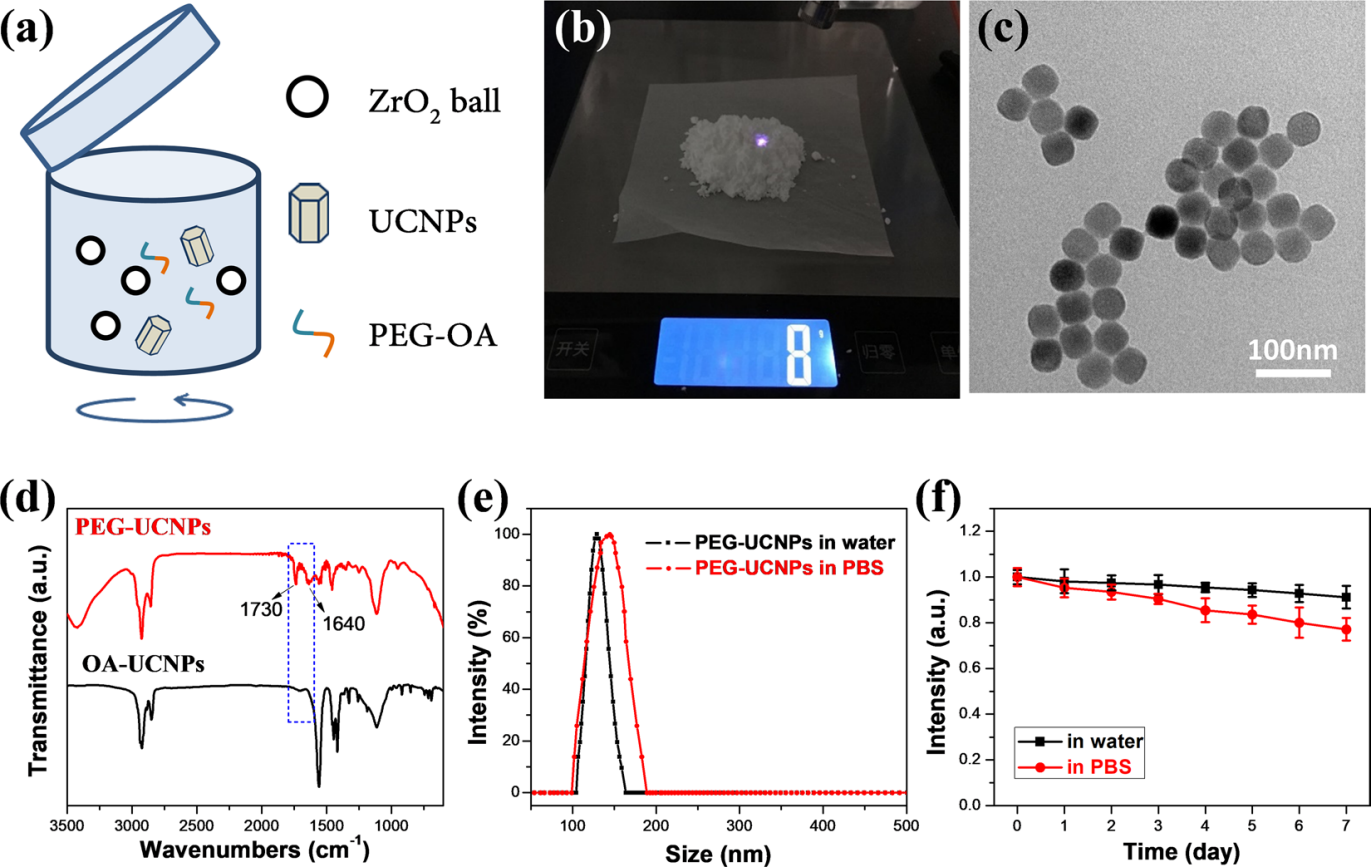
(**a**) Schematic illustration of the novel physical grinding method for the OA-PEG coating. (**b**) The total weight of final products was about 8 grams. (**c**) TEM image of PEG-UCNPs. (**d**) FT-IR spectra of OA-UCNPs and PEG-UCNPs. (**e**) DLS analysis of PEG-UCNPs dispersed in water and PBS. (**f**) Upconversion emission intensity of PEG-UCNPs in water and PBS as a function of days.

As shown in Fig. 3a, a certain amount of hydrophobic UCNPs, PEG-OA and Zirconium oxide (ZrO_2_) balls were mixed together and added to the agate jar for ball mill. Through mechanical ball-milling, the amphiphilic PEG-OA were absorbed onto the surface of UCNPs by hydrophobic interaction between OA chains, thus rendering UCNPs water dispersibility due to the hydrophilic PEG chains outside. The most attractive feature of this grinding strategy was to realize mass-production (Fig. 3b). The amount of the obtained product was only dependent on the size of the ball-milling jar while the yield and quality of traditional method were highly affected by the concentration of nanoparticles. The TEM image in Fig. 3c showed that a layer of polymer was coated on the surface of the nanoparticles. Next, Fourier transform infrared (FT-IR) spectra showed the changes of molecular bond on the surface of UCNPs, and confirmed the successful coating. As shown in Fig. 3d, the vibrations of -COOH at 1730 cm^−1^ and –OH at 1640 cm^−1^ were remarkably enhanced after modification, verifying the existence of PEG chain. Dynamic light scattering (DLS) measurements in Fig. 3e showed that the hydrodynamic radius of PEG-UCNPs was about 130 nm in pure water. Even in physiological solution, PEG-UCNPs exhibited good dispersibility with no apparent aggregation. High aqueous dispersion stability of PEG-UCNPs in pure water and PBS was also confirmed. The UCL intensity of the supernatant had a mild decrease after settling for 7 days, especially in PBS, indicating the slight aggregation and sedimentation (Fig. 3f and S11). The above results indicated that physical ball grinding should be an effective method to obtain high-quality water-dispersable UCNPs. Breaking the limit of low productivity of traditional liquid-phase sonication method, it has the potential to be applied in pilot scale test with the advantages of simplicity, flexibility and high-throughput.

### Preparation and characterization of photosensitizer-loaded UCNPs

**Figure 4:**
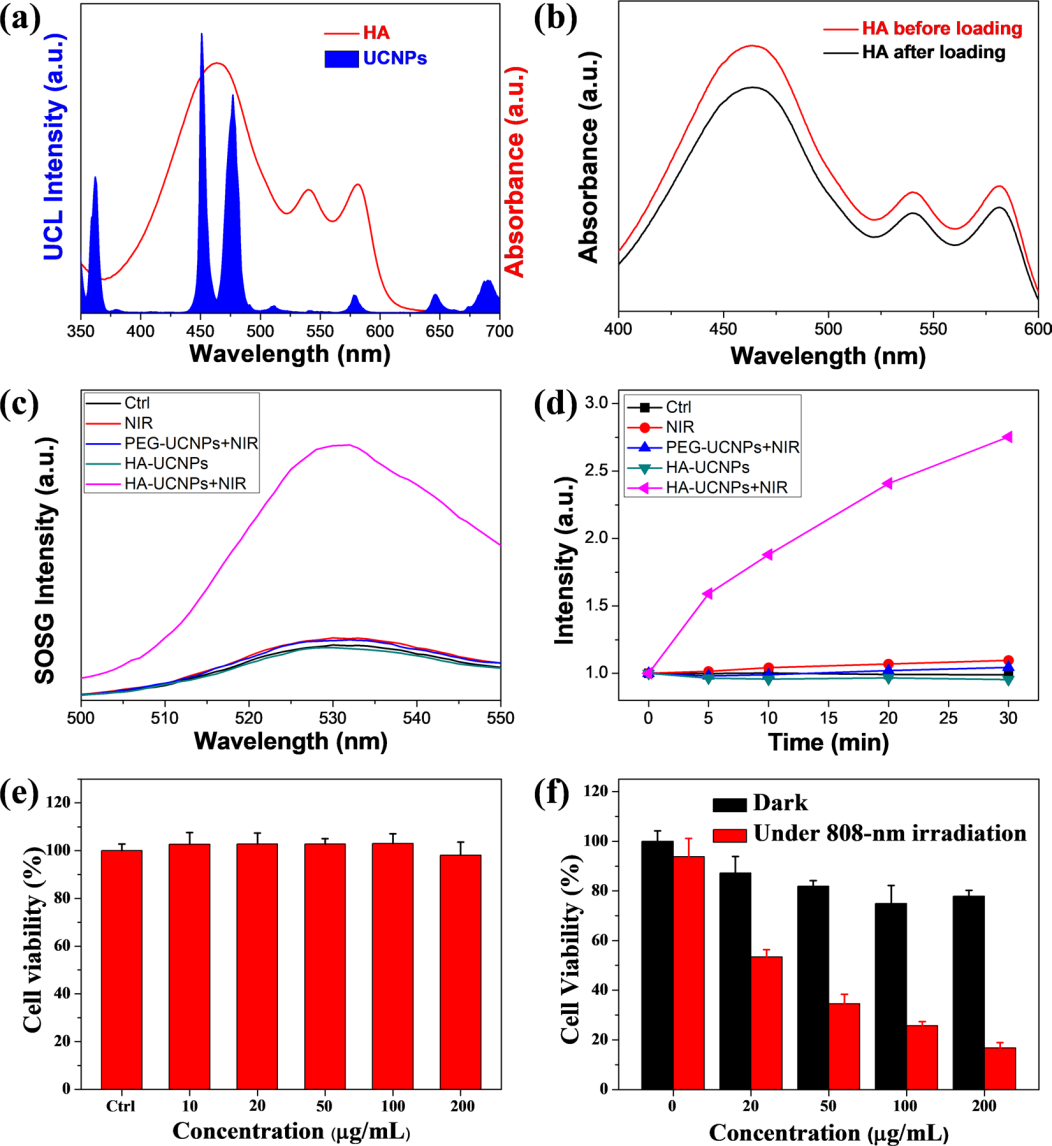
(**a**) UCL spectrum of UCNPs and UV-Vis absorption spectrum of HA in DMSO. (**b**) UV-vis spectra of HA before and after loading. (**c**) Fluorescence intensity of SOSG in different groups. (**d**) Emission intensity of SOSG at 525 nm in different groups. (**e**) Cell viability of BEL-7402 cells incubated with different concentrations of PEG-UCNPs for 24 h. (**f**) 808-nm laser induced PDT effect was evaluated on BEL-7402 cells. The data in (**e**) and (**f**) are shown as mean value and standard deviation, n = 3.

UCNPs have been widely used in photodynamic therapy (PDT) to increase the tissue penetration depth due to the unique properties of near-infrared (NIR) light^49–54^. Our synthesized UCNPs offered a strong blue UCL emission upon 800-nm irradiation, which well matched the main absorption of photo-sensitizer HA (Fig. 4a). The oleic acid chain of PEG-OA offered a hydrophobic domain for storage of water-insoluble drugs. Utilizing these properties, we construct a photodynamic therapeutic platform based on PEG-UCNPs and HA. Through the hydrophobic interaction, HA could be easily loaded onto the OA chains by simply mixing. As shown in Fig. 4b, the UV-Vis absorbance of HA declined a little after stirring with PEG-UCNPs, and the loading ratio was calculated to be about 5% (wt%). The stability of HA-UCNPs complex was also evaluated in physiological solution. There was nearly no obvious leakage of HA from these nanocomplex during 24 h stirring (Fig. S12). The emission of HA-UCNPs had no significant difference after 24 h stirring in water or PBS (Fig. S13), indicating the good stability of the obtained nanocomplex.

Next, cytotoxic singlet oxygen (^1^O_2_) generated from HA-UCNPs complex under 808-nm irradiation was measured by a commercial probe singlet oxygen sensor green (SOSG). SOSG could recognize ^1^O_2_ specifically and emit strong green fluorescence around 525 nm (Fig. 4c). In our experiments, HA-UCNPs (or PEG-UCNPs) were mixed with the same amount of SOSG in water and irradiated under 808-nm at different time intervals. As demonstrated in Fig. 4d, only the sample of HA-UCNPs complex under 808-nm irradiation showed a remarkable fluorescence increase, confirming the generation of ^1^O_2_ from this nano-complex. These results verified the success of constructing an 808-nm stimulated PDT platform based on the as-prepared PEG-UCNPs and HA, which could be further applied in cancer therapy.

### Photodynamic therapy *in vitro*

Encouraged by the efficient generation of ^1^O_2_ from HA-UCNPs complex, we evaluated the photodynamic effect on human hepatocellular carcinoma BEL-7402 cells. Firstly, the cytotoxicity of PEG-UCNPs was determined by CCK-8 assay. As shown in Fig. 4e, PEG-UCNPs exhibited no significant cytotoxicity during 24 h incubation even the concentration reached 200 μg mL^−1^. Next, the PDT effect was evaluated in dark condition to avoid the environmental disturbance. The viability of BEL-7402 cells without NIR irradiation remained above 80%, suggesting low dark toxicity of HA-UCNPs complex. On the contrary, the cell viability decreased significantly after irradiation under 808-nm laser, which confirmed the efficient PDT killing effect of newly-synthesized nanocomposite (Fig. 4f). Besides, we verified that there was no obvious heat effect during 808-nm irradiation (Fig. S14), which confirmed that it was ^1^O_2_ that damaged cellular organs and caused the death of tumor cells. To visually display the PDT effect, the live-dead staining was adopted after PDT treatment. The survival cells were stained with Calcein-AM (green fluorescence) while the nucleuses of dead cells were stained with Propidium Iodide (red fluorescence). From Fig. S15, nearly half of cells were dead after treated with 50 μg mL^−1^ of HA-UCNPs under 808-nm irradiation, and almost no cell survived when the concentration of HA-UCNPs increased to 100 μg mL^−1^ under 808-nm laser irradiation. Taken the above results together, our 808-nm PDT system based on HA-UCNPs was a promising efficient PDT agent for cancer therapy.

### Bio-imaging

**Figure 5:**
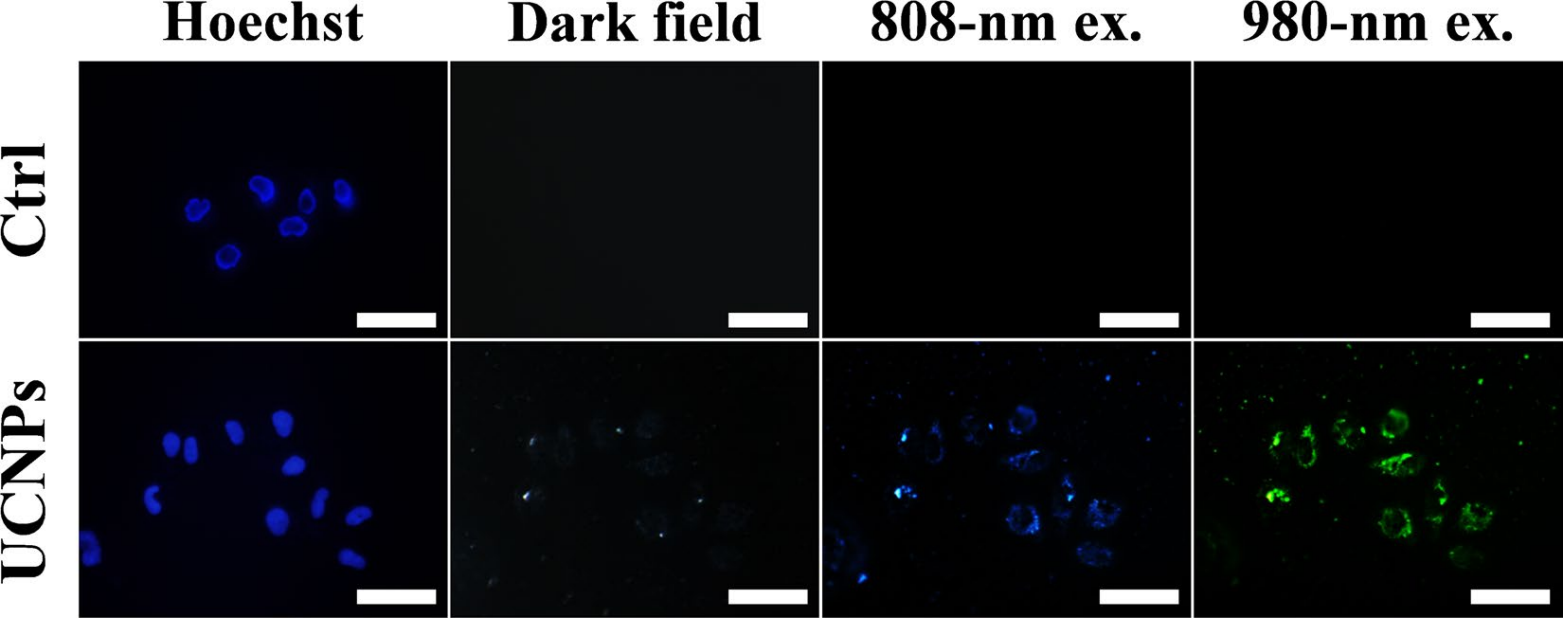
Cell UCL imaging using an inverted microscope equipped with 808-nm and 980-nm lasers. The nucleus was stained with Hoechst 33342. All the scale bars are 50 μm. The concentration of PEG-MS-UCNPs was 50 μg mL^−1^.

Owing to the unique optical properties, UCNPs have big potential to serve as fluorescent probes in the field of bio-imaging^55–60^. The practical application of UCL imaging was investigated using an inverted microscope equipped with both 808-nm and 980-nm laser. As shown in Fig. 5, after co-incubated with PEG-MS-UCNPs, BEL-7402 cells exhibited strong blue fluorescence under 808-nm irradiation and intense green fluorescence under 980-nm irradiation. With the increased concentration of UCNPs, the intensity of UCL fluorescence enhanced obviously (Fig. S16). More importantly, the signal-to-noise ratio of UCL imaging was much higher than the dark-field imaging. This feature made UCNPs a powerful probe to track small molecules and investigate the interactions between biomolecules and materials.

## Conclusion

In summary, we have successfully established a high-throughput strategy to fabricate gram-scaled water-dispersible upconversion nanoparticles. Utilizing the soluble Ln-OA precursors, we have also synthesized core nanoparticles and then fabricated core-shell structured UCNPs through SLBL strategy. The well-designed core-shell structures endowed UCNPs with enhanced upconversion fluorescence, tunable excitation wavelength and orthogonal excitations-emissions properties. To apply in biomedical field, the gram-scaled poly(ethylene glycol) monooleate-coated UCNPs were obtained via physical grinding method. Owing to the strong upconversion emission, the as-prepared UCNPs were further applied for bio-imaging, and could be also combined with HA to construct a PDT platform for cancer therapy.

## Methods

### Materials and reagents

Ln nitrate hydrates (Ln(NO_3_)_3_∙6H_2_O, 99.9%, Ln: Y, Yb, Nd, Tm, Er), Ln oxides (Ln_2_O_3_, 99.9%, Ln: Y, Yb, Tm), Ln chloride hydrates (LnCl_3_∙6H_2_O, 99.9%, Ln: Y, Yb, Tm), Sodium oleate (97.0%) and trifluoroacetic acid (TFA) were provided by Aladdin Chemical Co., Ltd. 1-octadecene (ODE, 90%) was obtained from Alfa Aesar Ltd. Poly(ethylene glycol) monooleate (PEG-OA, average molecular weight ~860) and oleic acid (OA, 90%) were supplied by Sigma-Aldrich. Sodium fluoride (NaF), ethanol, cyclohexane and dimethylsulfoxide (DMSO) were purchased from Beijing Chemical Reagent Company. Hypocrellin A (HA, 98%) was supplied by Shanghai Standard Biotech Co., LLC. Calcein-AM, propidium iodide and cell counting kit-8 (CCK-8) was provided by Dojindo Laboratories. Singlet oxygen sensor green (SOSG) was obtained from Invitrogen. All of the chemicals were analytical grade and used without further purification. Deionized (DI) water was used throughout experiments.

### Precursor preparation

Ln-OA was prepared as the following procedures. Typically, 10 mmol of yttrium nitrate and 30 mmol of sodium oleate were dissolved in a mixture solution of 30 mL of DI water, 30 mL of ethanol and 60 mL of cyclohexane. After stirring at room temperature for 24 h, the mixed solution was moved into a separating funnel. The upper organic layer containing yttrium-oleate complexes was collected and washed with DI water and ethanol (20 mL: 30 mL) three times. The final complexes were obtained in a waxy solid form by rotary evaporation and then dissolved in a mixture of OA and ODE (20 mL: 30 mL) for further use. The other Ln-OA precursors (Yb, Nd, Tm, and Er) were prepared according to the same procedure.

### High-throughput synthesis of core UCNPs

In the typical synthetic route, 45 mmol of Y-OA, 15 mmol of Yb-OA, 0.3 mmol of Tm-OA and 240 mmol of NaF were added into a 1 L double-necked reaction flask. Then, the appropriate volume of OA and ODE were added, making sure that the total volumes of OA and ODE were up to 180 mL and 450 mL, respectively. The mixture was then heated to 115 °C to remove water and oxygen under vacuum with stirring for 1.5 h. After that, the reaction was heated up to 310 °C (20 °C min^−1^) under argon atmosphere, and then kept at the temperature for 2 h. When the reaction cooled to room temperature, the final product was collected by centrifugation (12 000 rpm, 3 min), and washed with cyclohexane and ethanol (1:1 v/v) for three times.

### Synthesis of core-shell structured UCNPs using SLBL strategy

Firstly, the shell precursor (e.g. 0.5 mmol of Y-OA) was heated to 110 °C under vacuum with magnetic stirring to remove water. Then, 2 mmol of NaF and 0.5 mmol of core UCNPs were added into a solution of 6 mL of OA and 15 mL of ODE. The solution was kept at 110 °C for 60 min to remove the oxygen and residual water under vacuum. Afterwards, the mixture was heated to 310 °C under Ar atmosphere. After the temperature kept stable, the shell precursors were slowly and successively injected into the reaction system using a peristaltic pump (1 mmol h^−1^). The total injection and reaction time was one hour. Finally, the core-shell UCNPs were centrifuged and washed with cyclohexane and ethanol (1:1 v/v) for three times.

The other different kinds of core-shell structured UCNPs were synthesized using the same methods except the composition and volume of shell precursors. The amount of precursor in each shell layer was 1 mmol.

### Surface modification using ball grinding mill

Gram-scaled surface modification procedure was based on the ball grinding mill. Firstly, 5 g of the as-prepared hydrophobic UCNPs was dispersed in 2 mL of cyclohexane and then mixed with 8 g of poly(ethylene glycol) monooleate. The mixture was transferred in the ball grinding mill (ZrO_2_ ball), and then grinded at a certain speed (200 rad min^−1^) for 1 hour. The final products were collected by centrifugation and washed with water for three times, and finally were lyophilized for further use.

### Fabrication of HA-UCNPs complex

In the typical procedure, 25 mg of PEG-UCNPs (NaYF_4_:Yb/Tm@NaYbF_4_@NaYF_4_:Yb/Nd) was dispersed in DMSO solution containing 500uM of HA in 20-mL brown vials. After stirring for 24 h at room temperature, dark red complexes were collected by centrifugation and washed by DI water for several times. The drug loading capability was calculated by the change of UV-vis absorbance of the HA supernatant before and after the absorption. The leaching of the photosensitizer was measured with following steps. 4 mL of PBS solution with 0.5 mg/mL HA-UCNPs was stirring continuously under dark condition. After centrifuged at 12000 r/min for 5 min, the clear solution was measured by UV-visible spectrophotometer and the precipitation was re-dispersed in PBS. This process was repeated several times in different time points.

### The stability of fluorescence

4 mL of Water/PBS solution with 0.5 mg/mL HA-UCNPs/PEG-UCNPs were stored in dark condition for 24 hours. The fluorescence spectra of those four solutions were measured on different time points in quartz cell.

### Singlet oxygen (^1^O_2_) detection

Herein, singlet oxygen sensor green (SOSG) was adopted as a fluorescence probe to detect the generation of ^1^O_2_. Firstly, 2.5 μM of SOSG were mixed with HA-UCNPs, and then the mixture was irradiated under 808-nm laser for different intervals. The sample in dark condition was set as control. The generation of ^1^O_2_ was measured via the increase of SOSG emission peak at 525 nm under 488-nm excitation.

### Cell culture

Human hepatoma cell line (BEL-7402) was kindly presented by Professor Zheng Wang from Cancer Hospital Chinese Academy of Medical Sciences. The cells were cultured in DMEM (Gibco) with 10% fetal bovine serum (FBS, Gibco), 100 U mL^−1^ penicillin and 100 μg mL^−1^ streptomycin at 37 °C and 5% CO_2_.

### *In vitro* cytotoxicity study of PEG-UCNPs

BEL-7402 cells were seeded in 96-well plates at a density of 5 000 cells per well and then incubated for 24 h. The fresh media containing different concentrations of PEG-UCNPs (0, 10, 20, 50, 100 and 200 μg mL^−1^) was added and the cells were incubated for another 24 h. The CCK-8 assay was used to evaluate the cell viability of each sample, and the absorbance was determined by a Thermo Multiskan MK3 reader at 450 nm. The experiment was repeated at least three times.

### PDT effect *in vitro*

The cells were seeded in 48-well plate at a density of 10 000 cells per well. After adherence, different concentrations of HA-UCNPs complex were added (20, 50, 100 and 200 μg mL^−1^) and incubated for 6 hours. The cells were irradiated under 808-nm laser (0.8 W cm^−2^) for 10 min. After 24 hours, the cell viability was measured using CCK-8 assay. The results were read by microplate reader. Besides, Calcein-AM/propidium iodide (CA-PI) staining was also used to evaluate PDT effect. The cells were treated according to the same protocol as the CCK-8 assay. After PDT, 0.5 mL of PBS containing CA-PI (Calcein-AM, 2 μM; PI, 4 μM) was added to each well, and the cells were incubated at 37 °C for 15 min. Finally, the fluorescence images were obtained using an inverted fluorescence microscopy (IX73, Olympus).

### Cell imaging of HA-UCNPs

BEL-7402 cells were seeded in quartz-bottom dishes at a density of 20 000 cells per dish, and incubated for 24 h. Subsequently, the cells were treated with different concentrations of PEG-OA modified multi-shell UCNPs (PEG-MS-UCNPS: 0, 10, 20 and 50 μg mL^−1^) for 6 h. The redundant UCNPs were removed by PBS washing. Then, the cells were fixed using 4% paraformaldehyde, and stained with Hoechst 33342. Finally, the cells were imaged under NIR excitation using an inverted fluorescence microscope equipped with 980-nm and 808-nm lasers.

### Characterization

The shape and size of the as-obtained UCNPs were characterized by a JEM-2100Plus transmission electron microscopy. High-angle annular dark field were measured by Tecnai G2 F20 U-TWIN. Phase and crystal structure of UCNPs were measured using a Bruker D8 Advance. The upconversion fluorescence spectra and quantum yield were measured on an Edinburgh FLS 980 spectrofluorometer which an 8 W 980 nm diode laser. The UV-visible absorption spectra were captured using a Hitachi U-3900 spectrophotometer. DLS were measured by Malvern Zetasizer Nano ZS90. IR spectra were performed on a Thermo Fisher iN10-IZ10 spectrometer. All of the photos were acquired by a Nikon D3100 digital camera.

## Supplementary information

Supplementary Information

## Data Availability

The datasets generated during and/or analysed during the current study are available from the corresponding author on reasonable request.
