# Supplementary material for: Mass production of poly(ethylene glycol) monooleate-modified core-shell structured upconversion nanoparticles for bio-imaging and photodynamic therapy
Source: Sci Rep. 2019 Mar 26;9:5212. doi: 10.1038/s41598-019-41482-w (PMC6435707; doi:10.1038/s41598-019-41482-w)
Supplement: Supplementary file 1 — Supplementary Information [file 41598_2019_41482_MOESM1_ESM.docx]

**Mass production of poly(ethylene glycol) monooleate-modified core-shell structured upconversion nanoparticles for bio-imaging and photodynamic therapy**

**Xingyuan Zhang**^1,2,+^**, Zhao Guo**^2,3,+^**, Xiao Zhang**^2,*^**, Linji Gong**^2,3^**, Xinghua Dong**^3^**, Yanyan Fu**^5,*^**, Qing Wang**^1,4,*^**, and Zhanjun Gu**^2,3^

^1^Institute of Nano Engineering, College of Civil Engineering and Architecture, Shandong University of Science and Technology, Qingdao, 266590, China

^2^Key Laboratory for Biomedical Effects of Nanomaterials and Nanosafety, Institute of High Energy Physics, Chinese Academy of Sciences, Beijing, 100049, China

^3^University of Chinese Academy of Sciences, Beijing, 100049, China

^4^School of Material Science and Engineering, Shandong University of Science and Technology, Qingdao, 266590, China

^5^State Key Lab of Transducer Technology, Shanghai Institute of Microsystem and Information Technology, Chinese Academy of Sciences, Shanghai, 200050, China

^*^profqwang@163.com; fuyy@mail.sim.ac.cn; zhangx89@ihep.ac.cn

^+^these authors contributed equally to this work


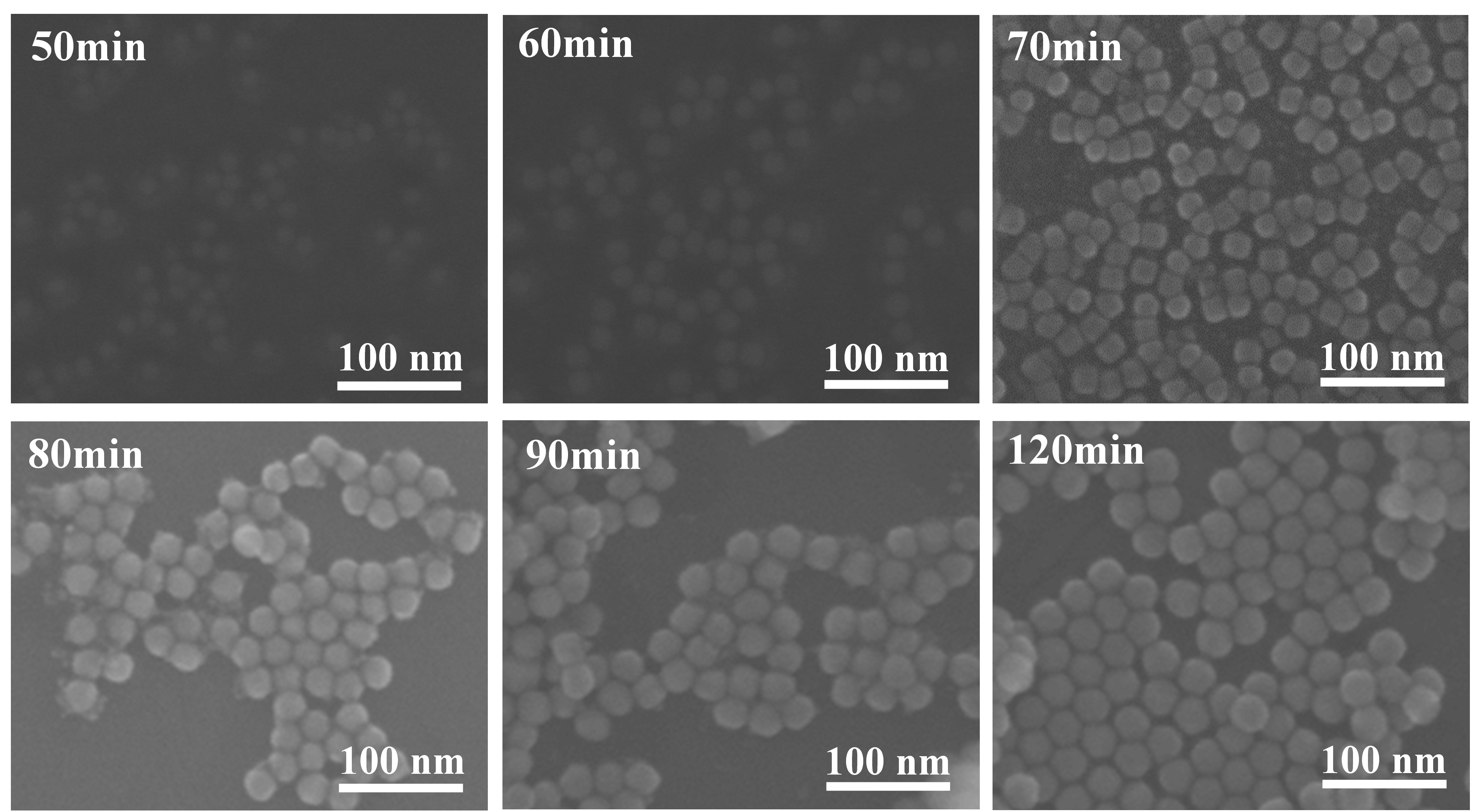


**Figure S1.** SEM images of NaYF_4_:Yb/Tm nanoparticles that collected after different reaction time.


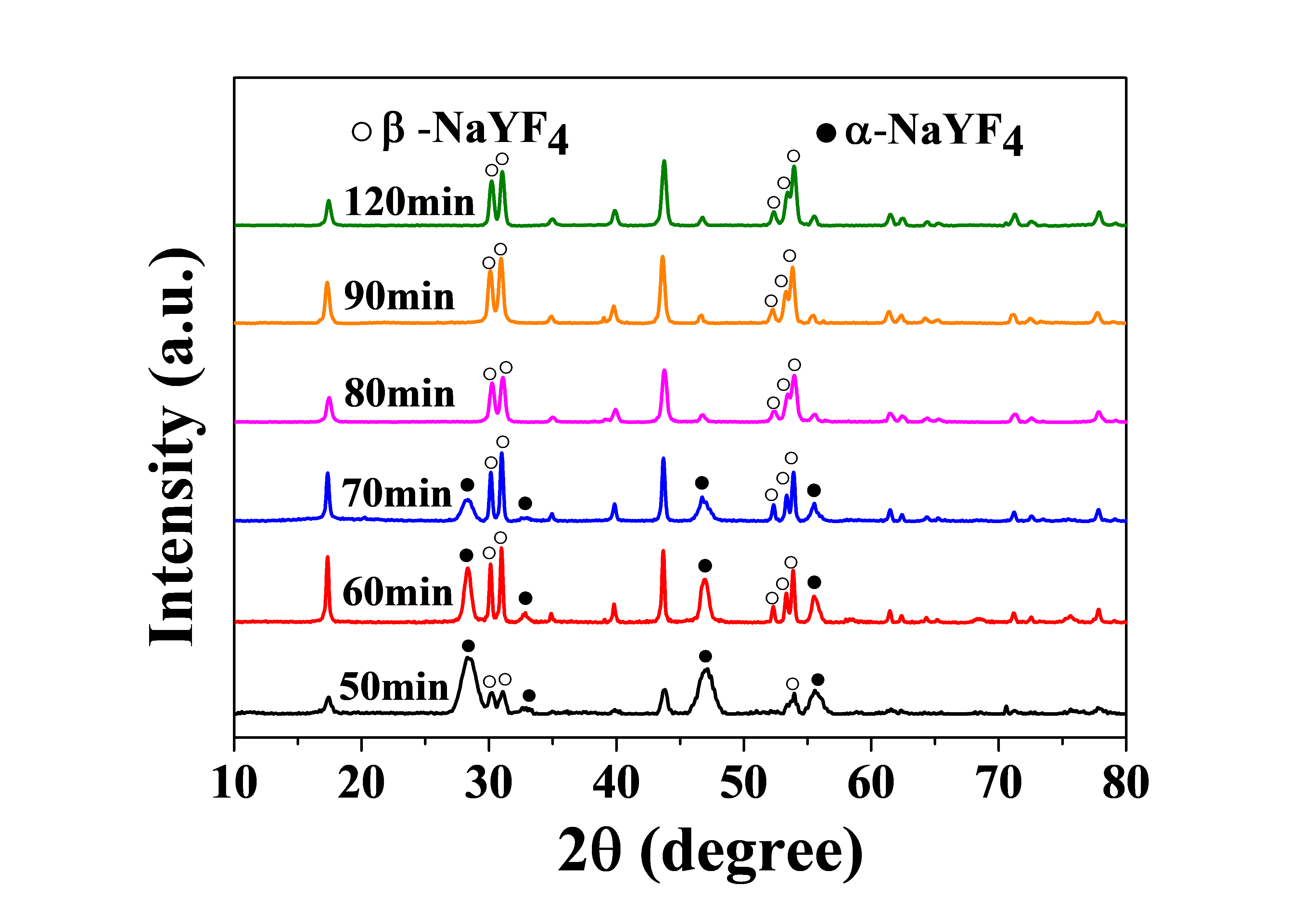


**Figure S2.** XRD patterns of NaYF_4_:Yb/Tm nanoparticles that collected after different reaction time.


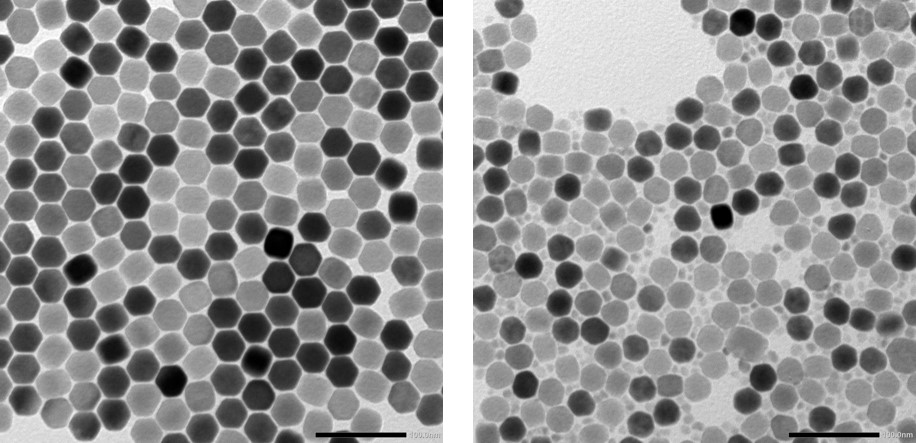


**Figure S3.** TEM images of UCNPs synthesized using LnCl_3_ as precursors with (left) or without (right) scavenging methanol. The scale bars are 100 nm.

**
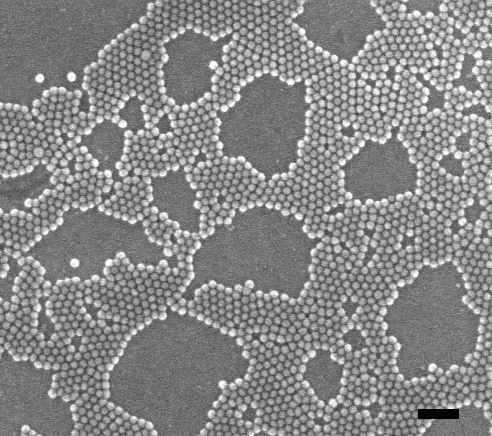
**

**Figure S4.** SEM images of Tm doped UCNPs synthesized at 290 ^o^C with the size of 20 nm. The scale bars are 100 nm.


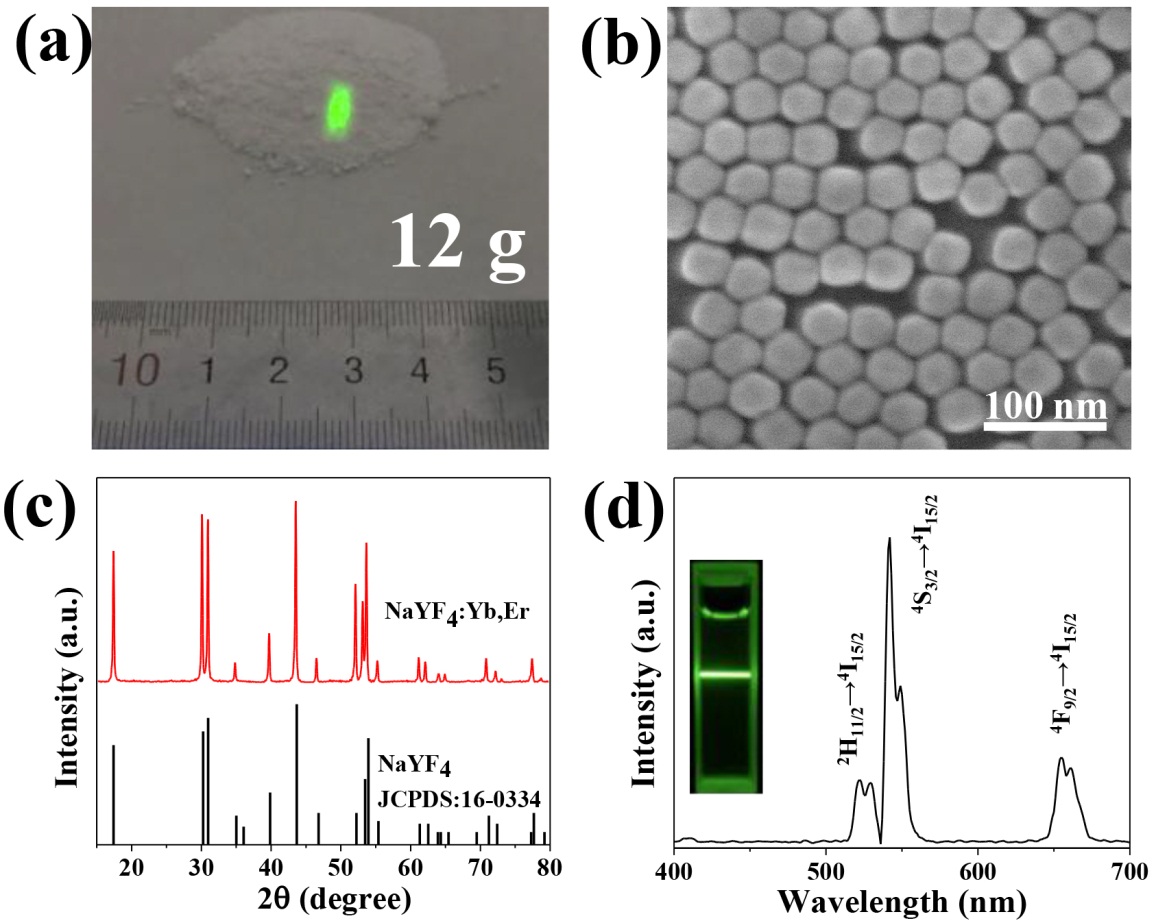


**Figure S5.** (a) The weight of the final products was about 12 grams. (b) SEM image of NaYF_4_:Yb/Er nanoparticles. (c) XRD patterns of the as-prepared UCNPs and standard β-NaYF_4_ card. (d) Fluorescence spectrum of UCNPs under 980-nm laser excitation. Inset: the UCL photograph.


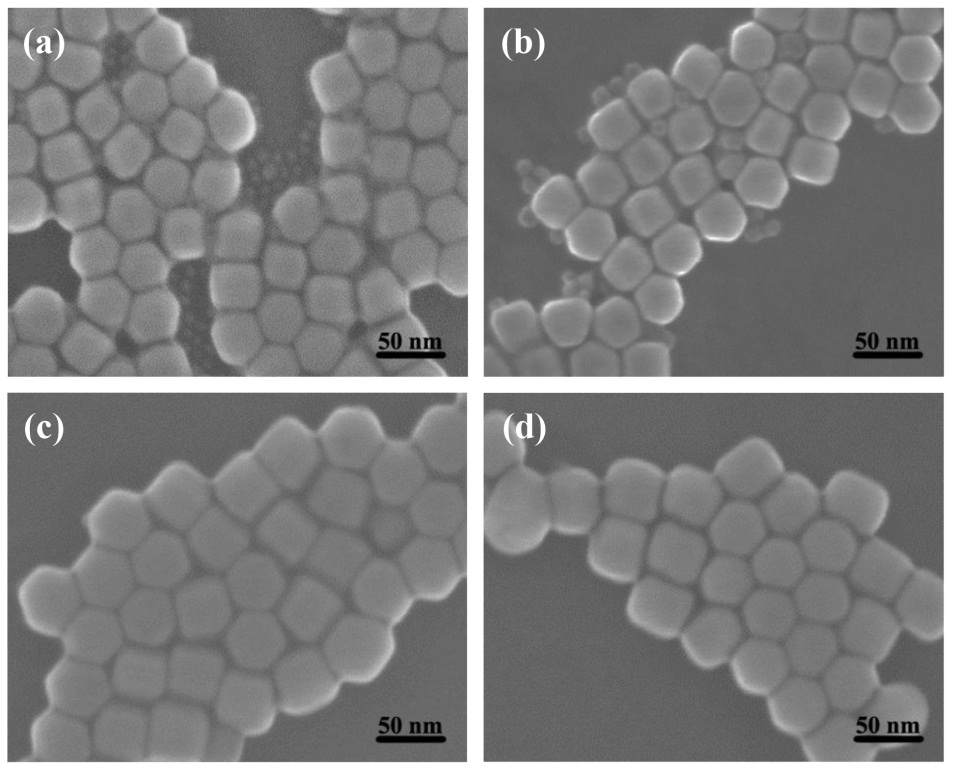


**Figure S6.** SEM images of CS-UCNPs with different reaction temperatures: (a) 290 ^o^C, (b) 300 ^o^C, (c) 310 ^o^C, and (d) 320 ^o^C.


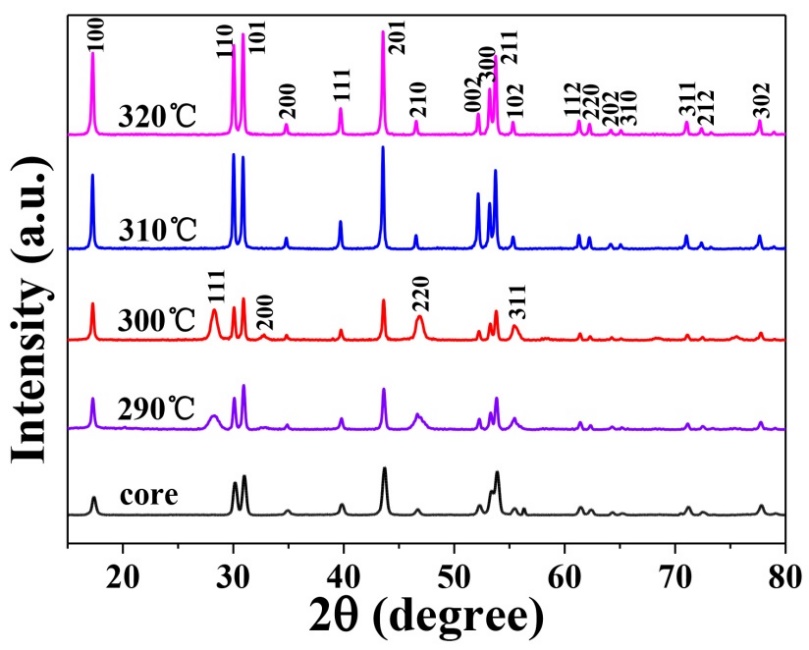


**Figure S7.** XRD patterns of the core NaYF_4_:Yb/Tm UCNPs and CS-UCNPs after reaction with different temperatures (290 ^o^C, 300 ^o^C, 310 ^o^C, 320 ^o^C).


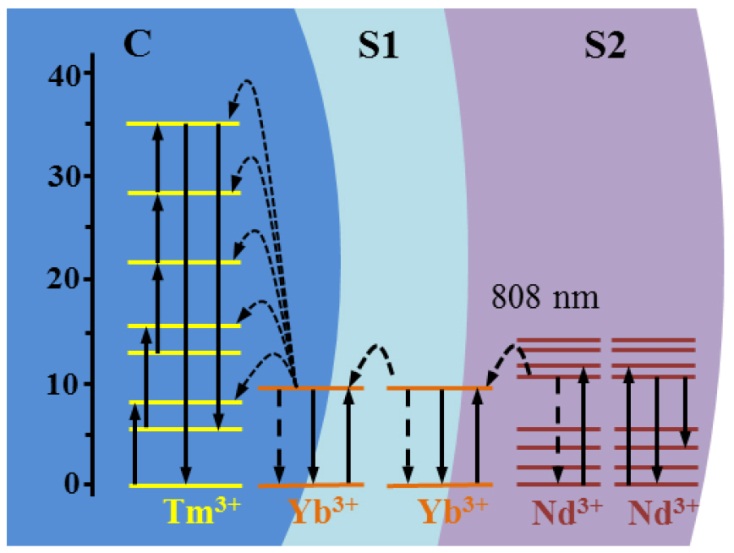


**Figure S8.** Energy-transfer mechanism of CSS-UCNPs under 808-nm irradiation.


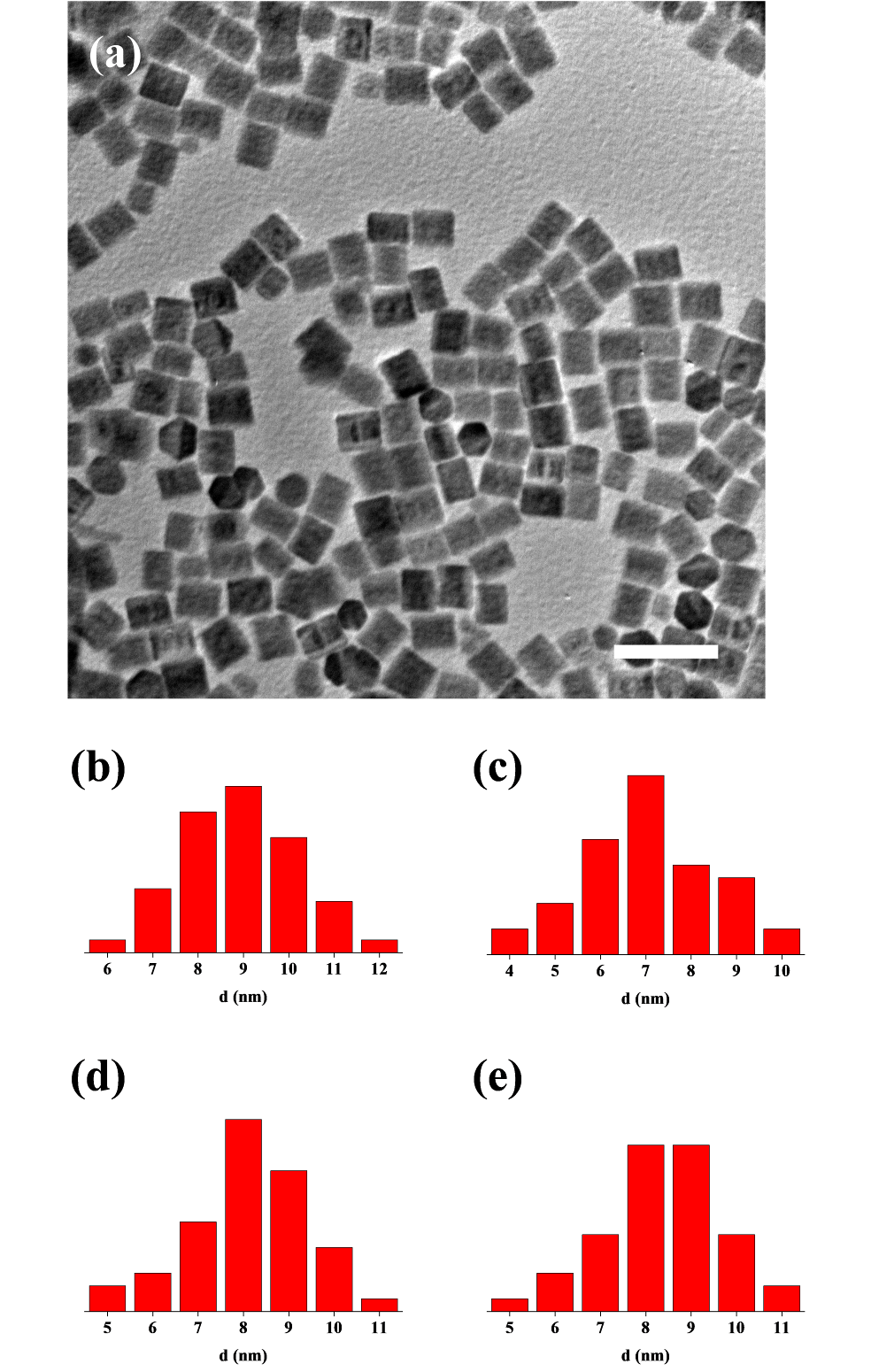


**Figure S9.** (a) TEM image of large area MS-UCNPs. The thickness of each shell, (b) shell 1: NaYF_4_:Yb/Nd, (c) shell 2: NaYF_4_, (d) shell 3: NaYF_4_:Yb/Er, (e) shell 4: NaYF_4_.

**
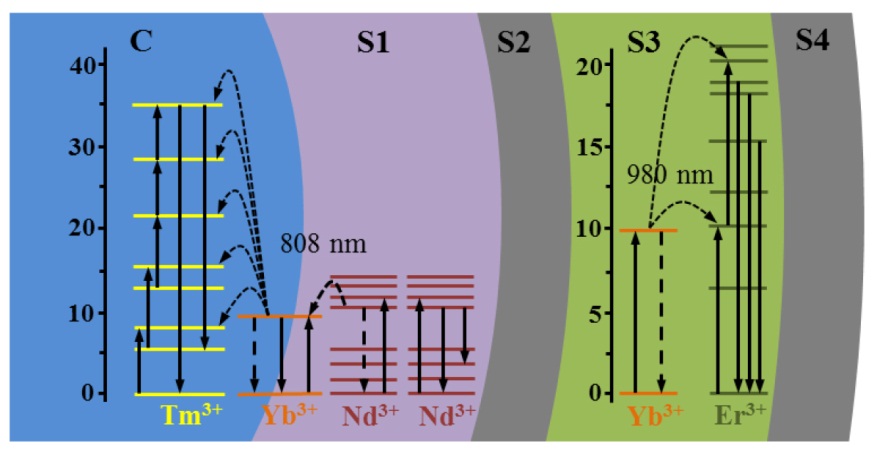
**

**Figure S10.** Energy-transfer mechanisms of the as-prepared MS-UCNPs under 808-nm or 980-nm irradiation.

**
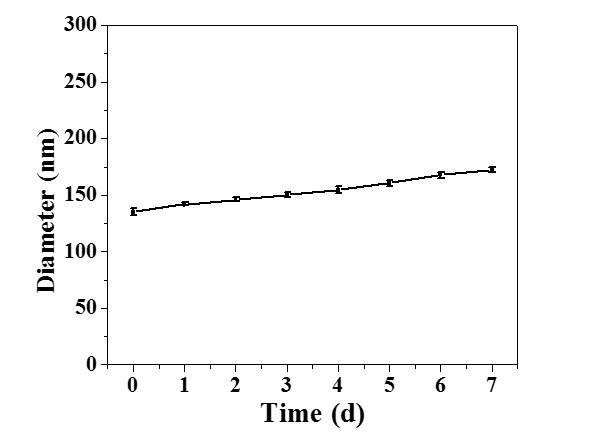
**

**Figure S11.** Hydrodynamic radius of PEG-UCNPs in PBS by DLS analysis.


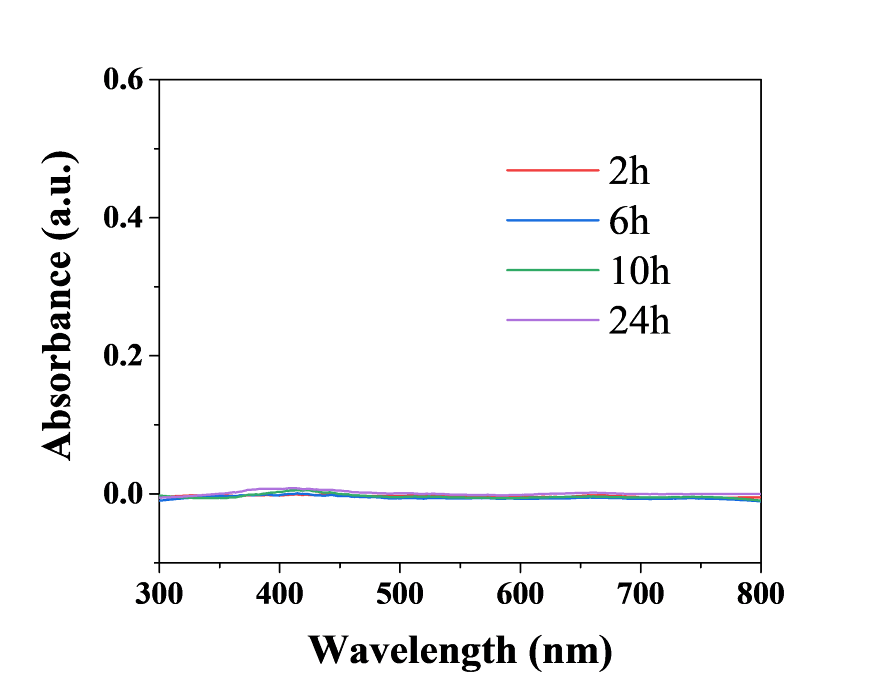


**Figure S12.** The UV-Vis absorption spectra of HA in the supernatant of HA-UCNPs complex in PBS solution.


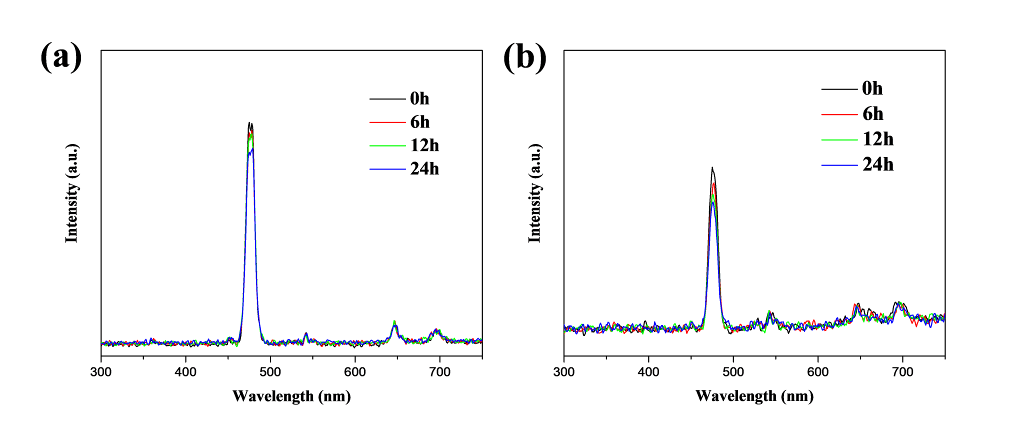


**Figure S13.** UCL emission spectra of HA-UCNPs with concentration of 0.5 mg mL in (a) water and (b) PBS after different hours stirring.


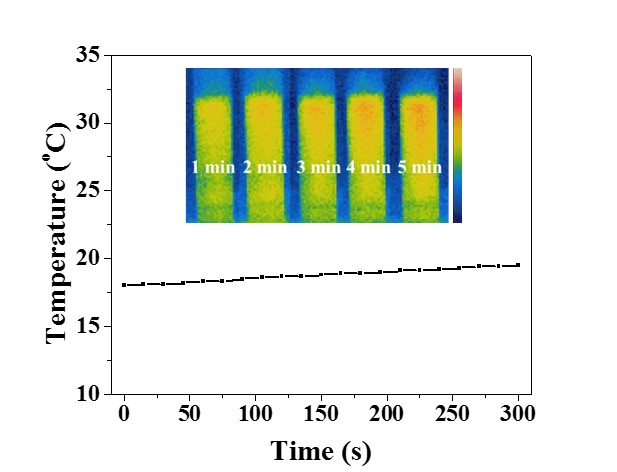


**Figure S14.** Temperature of PEG-UCNPs in aqueous solution under 808-nm irradiation. The laser power density and irradiation time are 0.8 W cm^-2^ and 5 min, respectively.


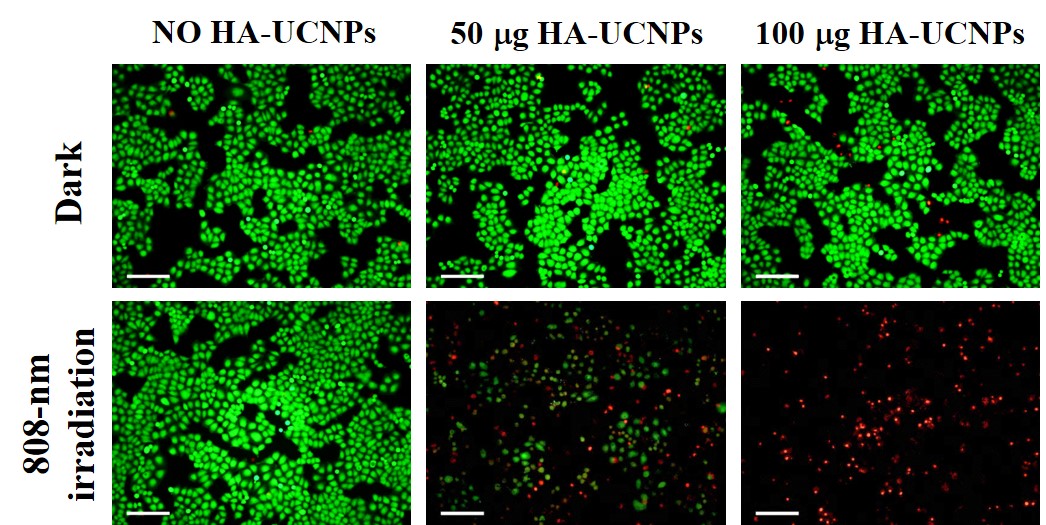


**Figure S15.** Fluorescent images of BEL-7402 cells after live–dead staining. The laser power density and irradiation time are 0.8 W cm^-2^ and 10 min, respectively. All the scale bars are 200 μm.


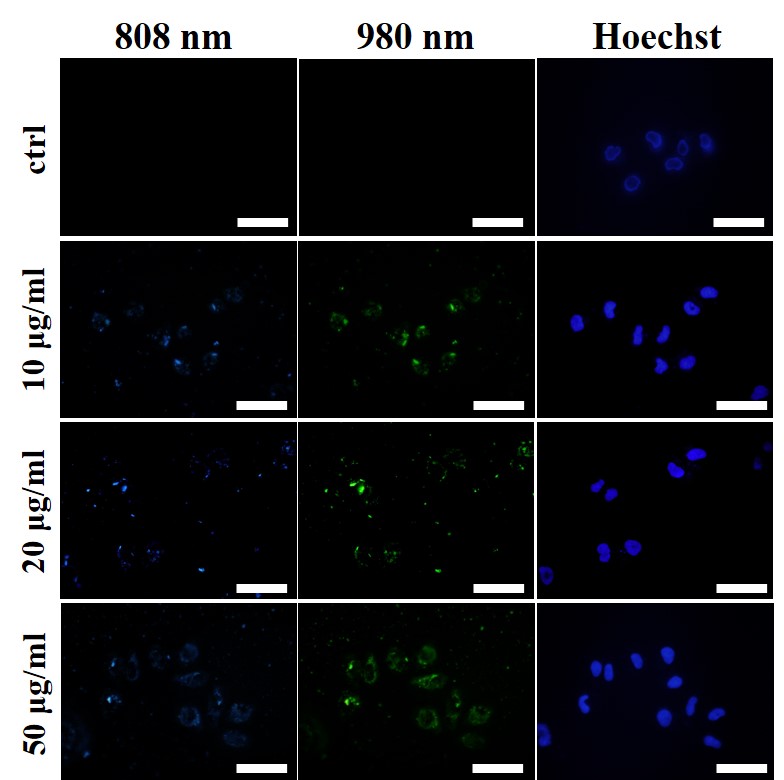


**Figure S16.** Cell UCL imaging using an inverted microscope equipped with 808-nm and 980-nm lasers. The nucleus was stained with Hoechst 33342. All the scale bars are 50 μm.
